# Feasibility of Radial Endobronchial Ultrasound-Guided Bronchoscopic Cryobiopsy without Fluoroscopy for Lung Parenchymal Lesions

**DOI:** 10.1155/2017/7170687

**Published:** 2017-11-15

**Authors:** Chih-Hao Chang, Chung-Shu Lee, Shih-Hong Li, Fu-Tsai Chung, Chih-Wei Wang, Yu-Hsiang Juan, Han-Chung Hu, Li-Fu Li, Ning-Hung Chen, Cheng-Ta Yang, Kuo-Chin Kao

**Affiliations:** ^1^Department of Pulmonary and Critical Care Medicine, Linkou Chang Gung Memorial Hospital, and Chang Gung University, Taoyuan, Taiwan; ^2^Division of Pulmonary and Critical Care, Department of Internal Medicine, Saint Paul's Hospital, Taoyuan, Taiwan; ^3^Department of Pathology, Linkou Chang Gung Memorial Hospital, Taiwan; ^4^Department of Medical Imaging and Intervention, Chang Gung Memorial Hospital, Linkou and Chang Gung University, Taoyuan, Taiwan

## Abstract

**Background:**

Cryobiopsy is used to biopsy peripheral lung lesions through flexible bronchoscopy with fluoroscopic guidance. However, fluoroscopy is not available at some institutions. This study evaluated the feasibility of radial endobronchial ultrasound-guided bronchoscopic cryobiopsy without fluoroscopy.

**Methods:**

This retrospective study was conducted at Chang Gung Memorial Hospital, Linkou branch, in Taiwan. This study enrolled patients who received bronchoscopy examinations with cryotechnology between July 2014 and June 2016. The data were collected through medical chart review.

**Results:**

During the study period, 101 patients underwent bronchoscopy examinations with cryotechnology. Ninety patients with endobronchial tumors were excluded from this study. Eleven patients who underwent radial endobronchial ultrasound-guided bronchoscopic cryobiopsy for lung parenchymal lesions were enrolled into this study. The mean age was 61.1 ± 13.8 years. Five patients were men, and the other six were women. The number of cryobiopsies ranged from 1 to 3. In the histological biopsies, the mean specimen diameter was 0.53 ± 0.23 cm, and the mean biopsy area was 0.20 ± 0.19 cm^2^. Nine of 11 patients had pathological diagnoses. No complications, including pneumothorax, respiratory failure, or major bleeding, were recorded after the procedure.

**Conclusions:**

Endobronchial ultrasound is used to ensure biopsy location, and endobronchial ultrasound-guided cryobiopsy is a feasible technique to biopsy peripheral lung lesions in selected cases at institutions without fluoroscopy equipment. This study provided some rationale for further studies examining the impact of fluoroscopy.

## 1. Introduction

Cryotherapy is an evolving therapeutic and diagnostic tool used in bronchoscopy [1]. Conventional forceps transbronchial biopsy (TBB) obtains a relatively small amount of alveolar tissue, and the specimen has more artifacts in the alveolar part [2]. Cryobiopsy has been recommended for diagnosing parenchymal lung diseases, including diffuse and peripheral lung lesions, because of its safety and high diagnostic yield rate [3, 4]. Studies have demonstrated that cryoprobe biopsy is an acceptable and useful technique for endobronchial mass diagnosis [5, 6]. Two recent systematic reviews have shown that cryotechnology in bronchoscopy is useful for the diagnosis of lung diseases, including interstitial lung diseases and lung tumors [7, 8]. A recent prospective, randomized, controlled, multicenter trial revealed a higher diagnostic yield for endobronchial cryobiopsy than for forceps biopsy [9]. For pulmonologists, the cryoprobe is a useful tool during flexible bronchoscopy examination.

The literature review revealed that fluoroscopic guidance was used in bronchoscopic cryobiopsy [3, 10–16]. However, the fluoroscopy equipment for bronchoscopy examination is not available at some institutions. During bronchoscopy examination, radial endobronchial ultrasonography (EBUS) is used to localize peripheral lung lesions, and the echoic feature can offer additional information on the structure of the lung lesions [17, 18]. To the best of our knowledge, the reports of cryobiopsy for peripheral lung lesions during flexible bronchoscopy are few. This retrospective study evaluated the feasibility of endobronchial ultrasound-guided cryobiopsy without fluoroscopy.

## 2. Method

### 2.1. Patients

This retrospective study was performed at Chang Gung Memorial Hospital, Linkou branch, a tertiary referral medical center in Taiwan. This study enrolled patients aged more than 20 years who received bronchoscopy examinations with cryotechnology between July 2014 and June 2016, and their data were collected through medical chart review. Patients with endobronchial mass who received cryotherapy through flexible bronchoscope were excluded. The clinical data of the study patients, including age, sex, pathological diagnosis, specimen size, procedure-related complications (i.e., pneumothorax, major bleeding, or respiratory failure), and outcomes, were retrospectively reviewed and analyzed.

### 2.2. Procedure and Equipment

Bronchoscopy was performed using a flexible bronchoscope (BF-P240 or BF-40; Olympus, Tokyo, Japan), and during the procedure, patients received local anesthesia with 2% xylocaine. A 20 MHz miniature radial probe (UM-S20-S20R; Olympus) and an ultrasound unit (Endoscopic Ultrasound System, Olympus) were also used. All patients underwent chest computed tomography (CT) before the bronchoscopy procedure, and the radial EBUS probe was inserted into the target segmental bronchus determined by the bronchoscopists. The echoic features of the peripheral parenchymal lesions were recorded [19]. Patients received conscious sedation with midazolam or propofol infusion under bispectral index for consciousness monitoring [20]. The heart rate, blood pressure, and oxygen saturation were continuously monitored during the bronchoscopy procedure.

### 2.3. Cryobiopsy Procedure

Cryobiopsy was performed using a 1.9 mm flexible cryoprobe (Erbokryo CA, Erbe, Germany) with carbon dioxide as the cryogen. A temperature of approximately −70°C was achieved at the probe tip. The biopsy sites were determined by the bronchoscopists according to the CT and EBUS findings. The probe cooling time was approximately 4–6 seconds. After cooling, the cryoprobe was immediately retracted using the bronchoscope. The frozen biopsy specimens were then thawed in normal saline and fixed in formalin. The bronchoscope was reintroduced to confirm airway status [5]. The size and diagnosis of the specimens were assessed by adequately experienced pathologists.

### 2.4. Statistical Analyses

Continuous variables are expressed as the mean ± standard deviation and categorical variables as the frequency and percentage. All statistical analyses were performed using MedCalc, version 12.5 (MedCalc Software, Ostend, Belgium). Two-tailed *p* values less than 0.05 were considered statistically significant.

## 3. Results

**Figure 1:**
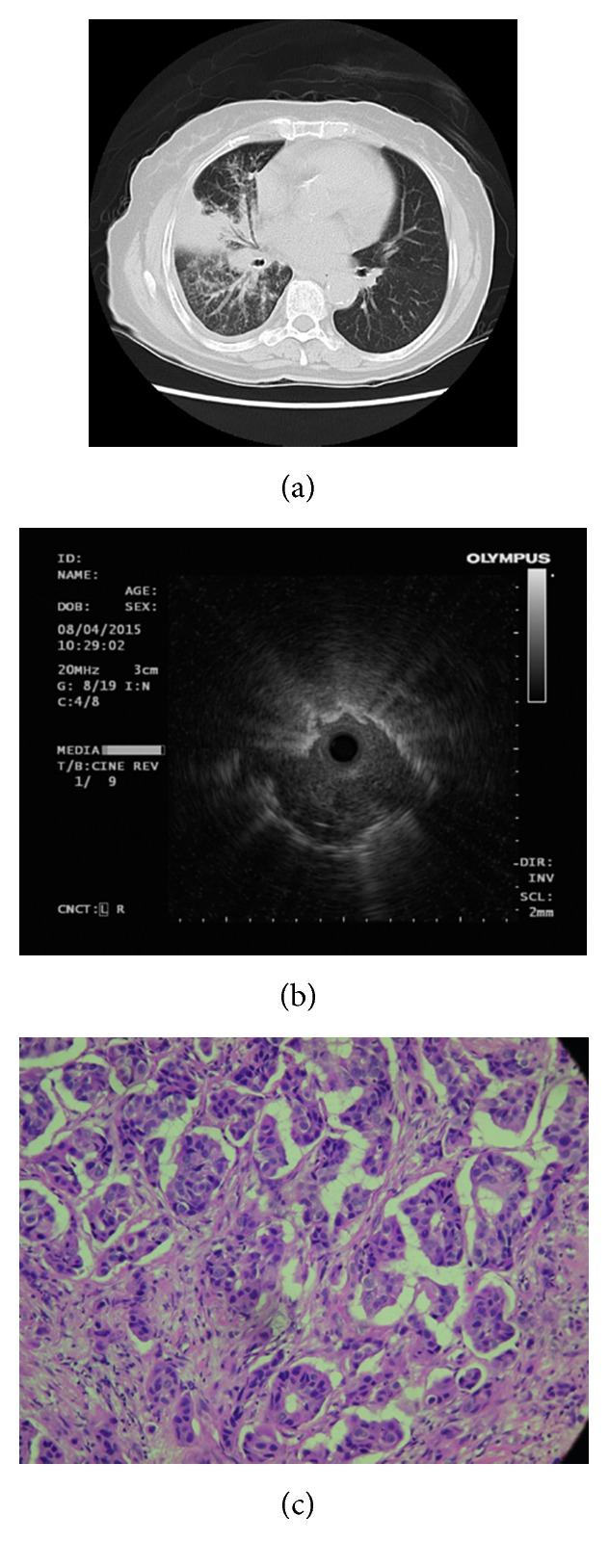
A 73-year-old female received cryobiopsy from right middle lobe showing correlation among the axial CT image, radial endobronchial ultrasound (EBUS) image, and histopathologic finding from biopsy. (a) Axial CT image in lung window demonstrated a right hilar mass with partial obstructive pneumonitis and numerous tiny ipsilateral lung nodules. (b) EBUS showed a heterogeneous echogenicity lesion with a continuous margin, and the probe was within the lung lesion (eccentric radial EBUS image). (c) Histologic specimen of the biopsy showed invasive nests of adenocarcinoma (hematoxylin and eosin staining, 200x).

**Figure 2:**
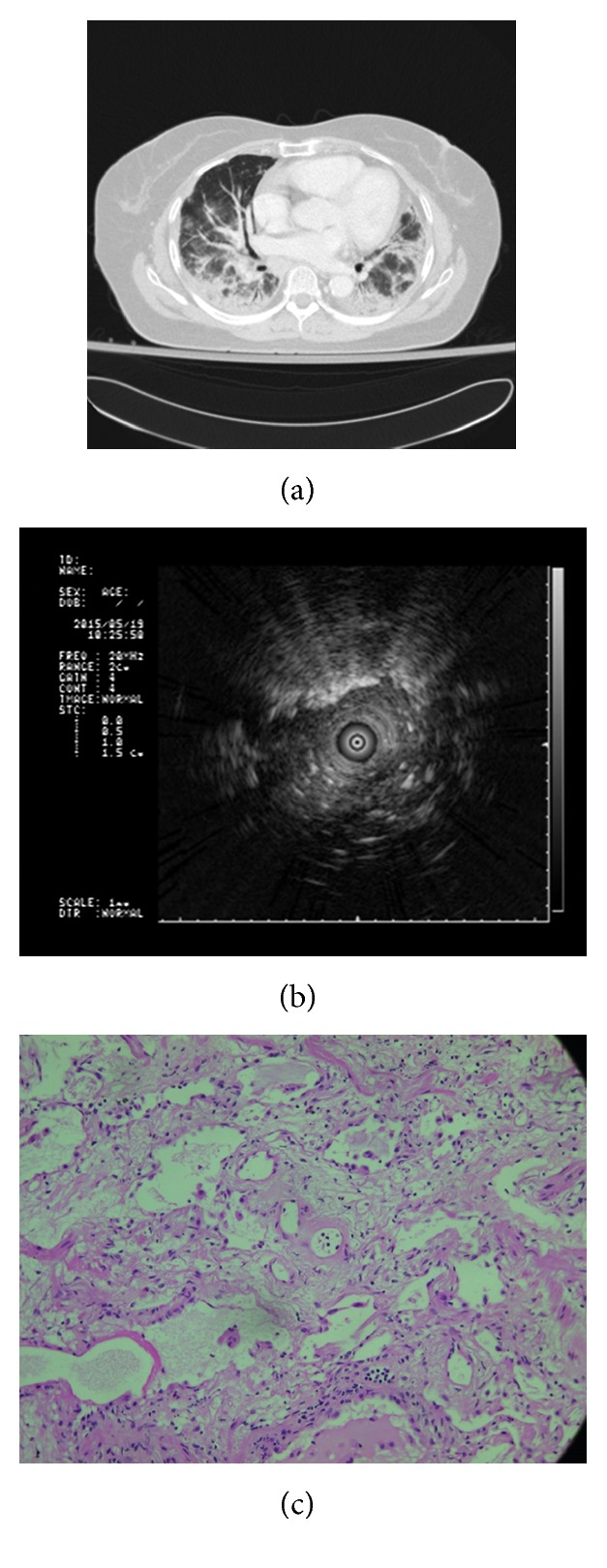
A 66-year-old female received cryobiopsy from lingula showing correlation among the axial CT image, endobronchial ultrasound (EBUS) image, and histopathologic finding from biopsy. (a) Axial CT image in lung window demonstrated ground-glass opacities in peribronchial distribution and dependent atelectasis in the bilateral dependent lung. The image appearance is nonspecific with several possible differential diagnoses, including atypical pneumonia, acute interstitial pneumonitis, nonspecific interstitial pneumonitis, and/or pulmonary edema. (b) EBUS showed heterogeneous echogenicity, along with linear–discrete air bronchogram. (c) Histologic specimen of the biopsy revealed interstitial homogenous fibrosis and chronic inflammation (hematoxylin and eosin staining, 200x).

During the study period, 101 patients underwent bronchoscopy examinations with cryotechnology. Ninety patients with endobronchial tumors were excluded from this study. Thus, this study included 11 patients with a mean age of 61.1 ± 13.8 years (Table 1). Five (45%) patients were men, and the other 6 were women. Six patients received bronchoscopy examination for diffuse interstitial lung disease, and the other five were for peripheral parenchymal lesions, respectively (Figures 1 and 2). Forceps biopsies were not applied simultaneously during the procedure. The six patients with diffuse interstitial lung disease received bronchoalveolar lavage. The other five patients with peripheral parenchymal lesions also received bronchial washing, and malignant cytology results were found in two cases. All the eleven patients did not receive endotracheal tube intubation during and after the procedure.

The number of cryobiopsies ranged from 1 to 3. In the histological biopsies, the mean specimen diameter was 0.53 ± 0.23 cm, and the mean biopsy area was 0.20 ± 0.19 cm^2^. Two patients had no histopathological diagnosis (negative for malignancy), and the other nine patients had pathological diagnoses. The biopsy sites and pathological diagnoses are provided in Table 2. The two patients with negative for malignancy had lung tissue described in the histology report. But the patient with chronic inflammation had only bronchial mucosa with lymphocytic infiltrates in the histology report. No major complications, including pneumothorax, respiratory failure, admission to the intensive care unit, or major bleeding with the need for further intervention, were recorded. The final diagnosis of interstitial lung disease was made by the clinical physicians individually according to the clinical data, radiologic study, biopsy results, and follow-up data. The final diagnoses of the six patients with interstitial lung disease were two idiopathic pulmonary fibrosis, two nonspecific interstitial pneumonia, one sarcoidosis, and one undiagnosed. One of the two patients with pathological negative for malignancy was diagnosed with non–small cell lung cancer by CT-guided biopsy, and the other one remained undiagnosed because the patient did not receive a further examination and did not return to our hospital for follow-up.

## 4. Discussion

Few studies have used radial EBUS-guided cryobiopsy without fluoroscopy for peripheral lesions. An important finding of our study is that radial EBUS-guided cryobiopsy is useful to biopsy peripheral lung lesions at institutions without fluoroscopy equipment. The biopsy specimen retrieved was adequate, and no major complications were noted.

Cryotechnology is useful and safe for diagnosing and treating central airway lesions [21]. Fluoroscopy is useful for identifying peripheral lung lesions during the bronchoscopy procedure [22]. Fluoroscopy also ensures that the bronchoscope does not contact the visceral pleura, thus preventing the development of iatrogenic pneumothorax. In DiBardino et al.'s study, fifteen patients received transbronchial cryobiopsies without fluoroscopy, and two had pneumothorax [23]. Fluoroscopy is recommended to reduce the complications. However, fluoroscopy is not always available at many institutions. Therefore, radial EBUS is useful to biopsy peripheral lesions, instead of fluoroscopic guidance [24]. Although radial EBUS-guided transbronchial forceps biopsy without radiographic fluoroscopy is effective for the diagnosis of peripheral lung lesions [25], bronchoscopists are concerned about the complications of cryobiopsies, because of the large size of the specimen retrieved. To prevent pneumothorax development, some researchers have suggested that the cryoprobe should be withdrawn approximately 1 cm during cryobiopsy for diffuse lung disease. In our study, radial EBUS was used to localize peripheral lung lesions. If the radial probe reached the distal lung lesion, we could use the echoic feature to decide the optimal biopsy site. In diffuse lung disease, we could also avoid biopsy when the EBUS echoic feature showed prominent vessels. To select biopsy site by bronchoscopists is relatively subjective. Kropski and his colleagues suggested taking biopsies from the dependent part of the lungs in concern of bleeding risk [12].

The procedure of cryobiopsy through a flexible bronchoscope varies across studies, and the cooling time and the cryoprobe size have not been standardized [26]. In this study, a 1.9 mm cryoprobe was used, and the specimen size is similar to that described in the literature but is larger than the forceps biopsy specimen size [3]. A previous study suggested that two cryobiopsies are optimal for endobronchial tumors [27], but the optimal number of cryobiopsies for peripheral lesions has not been determined. Recently, important issues regarding safety and standardization of transbronchial cryobiopsies were addressed [23, 28]. The complications, including serious bleeding, iatrogenic pneumothorax, and respiratory failure, were reported. DiBardino et al. reported major bleeding in three patients, all of whom had undergone cryobiopsy without fluoroscopy [23]. There were no severe or life-threatening complications in our study. The possible explanations may be that our procedure was relatively conservative (using a 1.9 mm cryoprobe, taking biopsies in single lobe with few number of biopsies). Besides, all the patients had radial EBUS guided. Safety remains the major concern of the cryobiopsy procedure, and bronchoscopists should do a complete preprocedural evaluation, perform the procedure meticulously, and use all the available facilities, including fluoroscopy, at their hospitals. To optimize and standardize the transbronchial cryobiopsy procedure is also important for further studies.

The diagnostic yield rate of transbronchial biopsy is influenced by several factors, including lesion size, lesion location, bronchus sign, rapid on-site evaluation, prevalence of disease in the patient cohort, or navigation systems [25, 29–31]. The radial EBUS probe can easily access lesions close to the bronchial wall. In this study, patients did not receive surgical biopsy finally, and a multidisciplinary team did not decide the diagnosis of interstitial lung disease. The final diagnosis and treatment were decided by clinical physicians individually according to the imaging study and biopsy results. A systematic review showed a high diagnostic yield for transbronchial lung cryobiopsy when histopathology was combined with multidisciplinary discussion [32]. Recent studies have also shown that cryobiopsy is useful when interstitial lung disease is diagnosed through multidisciplinary discussion [33, 34].

Our study has several limitations. First, this study was conducted in a single tertiary referral medical center; thus, the generalizability of the findings may be limited. Bronchoscopists have a high level of experience for interventional bronchoscopy, including cryotechnology and transbronchial biopsy. Second, the study was retrospective, and the inclusion of patients receiving bronchoscopic cryobiopsy might be highly selective. We had no standard procedure protocol for patient selection. Third, the sample size was small, because transbronchial cryobiopsy was an alternative method to surgical biopsy for diagnosis of interstitial lung disease.

## 5. Conclusion

EBUS-guided cryobiopsy is a feasible technique to biopsy peripheral lung parenchymal lesions in selected cases to obtain a large specimen at institutions without fluoroscopy equipment. This study provided some rationale for further studies examining the impact of fluoroscopy.

## Figures and Tables

**Table 1:** Baseline characteristics of patients receiving cryobiopsy (*n* = 11).

Characteristic	Value
Age	61.1 ± 13.8
Sex (female), *n* (%)	6 (54.5)
Outpatient, *n* (%)	7 (63.6)
Body mass index	25.3 ± 6.7
Prebiopsy diagnosis	
Interstitial lung disease, *n* (%)	6 (54.5)
Peripheral parenchymal lesions, *n* (%)	5 (45.5)
Pulmonary function tests	
FEV1 percentage predicted (%)	63.9 ± 32.1
FVC percentage predicted (%)	61.9 ± 24.5

**Table 2:** Pathological diagnoses of patients receiving cryobiopsy (*n* = 11).

Pathology	Number
Biopsy location	
Right middle lobe	5
Right lower lobe	3
Left upper lobe	1
Lingula	2
Pathological diagnosis	
Adenocarcinoma	3
Interstitial fibrosis	5
Chronic inflammation	1
Negative for malignancy	2
